# OLDER ADULTS' PREPAREDNESS TO AGE IN PLACE: FINDINGS FROM THE NATIONAL POLL ON HEALTHY AGING

**DOI:** 10.1093/geroni/igac059.905

**Published:** 2022-12-20

**Authors:** Sheria Robinson-Lane, Erica Solway, Dianne Singer, Matthias Kirch, Jeffrey Kullgren, Preeti Malani

**Affiliations:** University of Michigan School of Nursing, Ann Arbor, Michigan, United States; University of Michigan, Ann Arbor, Michigan, United States; University of Michigan, Ann Arbor, Michigan, United States; University of Michigan, Ann Arbor, Michigan, United States; University of Michigan, Ann Arbor, Michigan, United States; University of Michigan, Ann Arbor, Michigan, United States

## Abstract

Though aging in place is a stated goal for many older adults, complications of advanced disease or disability can derail these plans. Little is known about the current preparedness of older adults to age in place. The January 2022 National Poll on Healthy Aging surveyed adults age 50-80 (n=2,277) about their home and available supports. Findings indicate that while 80% of adults believe they have the necessary features in their home to support aging in place, less than one in five had homes that were wheelchair accessible and fewer than half had other common safety and accessibility features. Older adults were most confident they could obtain help with household chores (51%) and were least confident about getting assistance with personal care (33%). Persons with disabilities (n=683) and those living alone (n=639) reported the most concerns. Several opportunities exist to better prepare older adults to successfully age in place.

